# Machine Learning Modelling, Single‐Cell Landscape Profiling and Spatial Transcriptomics Provide New Insights Into SUMOylation in Head and Neck Squamous Cell Carcinoma

**DOI:** 10.1049/syb2.70082

**Published:** 2026-07-29

**Authors:** Zhe Fang, Kai Mei, Jiaqi Liu, Wei Zhou, Tingjing Li, Hai Zhang, Chuangjie Cao

**Affiliations:** ^1^ Department of Oncology The First Affiliated Hospital of Hengyang Medical School University of South China Hengyang Hunan China; ^2^ School of Chinese Medicine Hunan University of Chinese Medicine Changsha Hunan China; ^3^ Department of Pathology The First Affiliated Hospital Hengyang Medical School University of South China Hengyang Hunan China

**Keywords:** cell cycle, head and neck squamous cell carcinoma, SAE1, SUMOylation

## Abstract

SUMOylation is implicated in the regulation of multiple malignancies. However, its potential roles in head and neck squamous cell carcinoma (HNSCC) remain insufficiently characterised. By integrating bulk RNA‐seq, scRNA‐seq and stRNA‐seq datasets, we systematically interrogated the biological relevance of SUMOylation in HNSCC. Key markers from the signature were further validated using in vitro functional assays. A recognition model was established and validated using 692 HNSCC and 178 non‐HNSCC samples. SROC analysis demonstrated robust performance across eight datasets (AUC = 0.92). Functional enrichment and scRNA‐seq analyses indicated that SUMOylation may exert its effects in HNSCC primarily through cell‐cycle regulation. Among the model features, *SAE1* was markedly overexpressed in HNSCC (SMD = 1.07, 95% CI 0.56–1.59, *p* < 0.05). In vitro assays further confirmed that *SAE1* enhanced proliferation, colony formation and migration in SAS and SCC‐9 cells and validated its role in regulating cell cycle progression and apoptosis. We established a SUMOylation‐related recognition model for HNSCC with consistently strong performance in multiple external validation cohorts. *SAE1* emerges as a candidate molecular biomarker for HNSCC.

## Graphical abstract

(1) Multi‐omics profiling reveals SUMOylation as a key oncogenic programme in HNSCC linked primarily to cell‐cycle regulation. (2) A SUMOylation‐based diagnostic model shows strong cross‐cohort robustness (692 HNSCC vs 178 non‐HNSCC; SROC AUC = 0.92 across 8 independent cohorts). (3) SAE1 is consistently overexpressed and functionally drives malignant phenotypes, promoting proliferation, colony formation and migration in HNSCC cells.

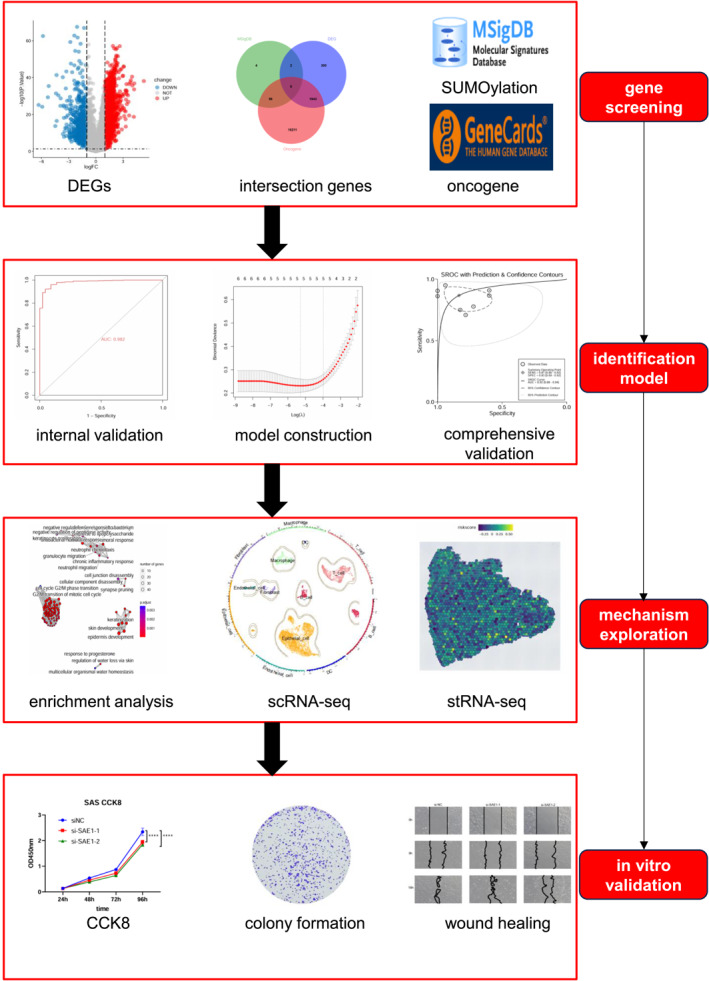

## 1 Introduction

Head and neck squamous cell carcinoma (HNSCC) is among the most prevalent malignancies of the head and neck region. In 2022, head and neck cancers accounted for approximately 0.94 million new cases and 0.48 million deaths worldwide underscoring a substantial and persistent global burden [1]. HNSCC frequently compromises swallowing, phonation and facial appearance; post‐treatment pain, dysphagia and impaired quality of life are common and closely linked to functional disability [2]. Despite multidisciplinary management, patients with locally advanced HNSCC remain at high risk of recurrence. Prior studies have reported recurrence rates of approximately 50% with treatment failure often occurring early, indicating that clinical outcomes remain suboptimal [3, 4]. Immunotherapy has improved survival for patients with recurrent or metastatic disease. In KEYNOTE‐048, first‐line pembrolizumab‐based regimens consistently demonstrated an overall survival advantage [5]. Nonetheless, real‐world cohorts highlight pronounced interindividual variability and uncertainty in therapeutic benefit [6, 7]. Moreover, robust and reproducible predictive biomarkers for recurrent or metastatic HNSCC are still lacking in routine clinical practice [8]. Spatial transcriptomic (stRNA‐seq) profiling has revealed distinct malignant cell states and interaction architectures between the tumour core and invasive margin, emphasising the marked intratumoral heterogeneity of HNSCC [9]. Collectively, these observations motivate deeper mechanistic interrogation and the identification of novel biomarkers for improved risk stratification and therapeutic decision‐making.

SUMOylation is a reversible post‐translational modification initiated by the E1 activating enzyme complex (*SAE1*/*UBA2*), followed by E2‐mediated conjugation and reversed by deSUMOylating enzymes such as SENPs; it has been increasingly recognised as a key contributor to oncogenic pathway rewiring [10]. Accumulating evidence across cancer types supports a functional role for SUMOylation in tumour regulation. In cholangiocarcinoma, upregulation of *SAE1* and *UBE2I* is accompanied by increased SUMO1‐conjugated proteins, and pharmacologic or genetic inhibition of SUMOylation promotes apoptosis, induces cell‐cycle arrest and attenuates tumour growth in vivo [11]. *UBE2I* has been shown to enhance SUMOylation of *hnRNPA2B1* and facilitate its cytoplasmic localization, thereby promoting thyroid cancer progression [12]. Therapeutically, TAK‐981, which targets *SAE1* and suppresses the SUMO pathway, markedly inhibits migration and clonogenicity in rhabdomyosarcoma cells [13]. In gastric cancer, a PIASxα‐driven p38α‐SUMOylation/MK2 axis forms a positive feedback loop with oxidative stress and has been proposed as a potential therapeutic vulnerability [14]. In contrast, systematic dissection of SUMOylation‐related mechanisms and biomarker discovery in HNSCC remains limited.

**FIGURE 1:**
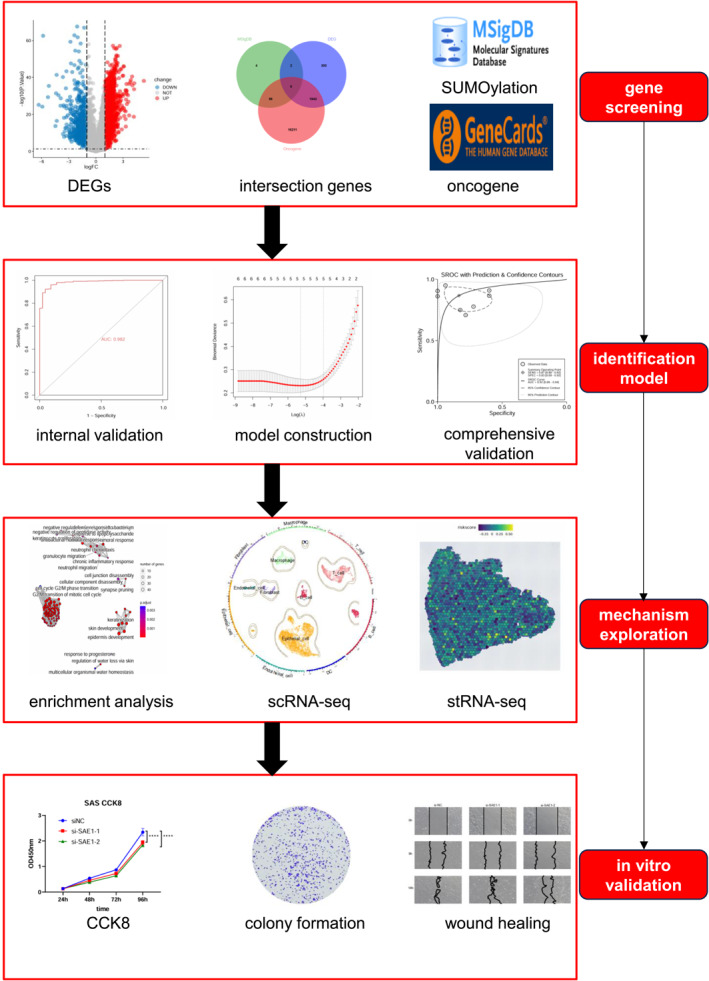
Flowchart of this study.

Here, we first developed a SUMOylation‐associated recognition signature for HNSCC. We then integrated bulk RNA‐seq, single‐cell RNA sequencing (scRNA‐seq) and stRNA‐seq data to comprehensively characterise the biological relevance of SUMOylation in HNSCC. Finally, we performed in vitro functional assays to validate the phenotypic effects of the key signature component. The main design of this study is detailed in Figure 1.

## 2 Materials and Methods

### 2.1 Collection of Multicenter Bulk RNA‐Seq Datasets

Bulk RNA‐seq datasets for HNSCC were retrieved from The Cancer Genome Atlas (TCGA) and the Gene Expression Omnibus (GEO). TCGA data were downloaded from the Xena browser, whereas GEO datasets were obtained using the GEOquery package. Corresponding clinicopathological variables were collected when available. The GEO cohorts included GSE107591, GSE33205, GSE58911, GSE59102, GSE53819 and GSE37991. Notably, GSE59102, GSE53819 and GSE37991 comprised laryngeal cancer, nasopharyngeal carcinoma and oral cancer tissues, respectively. Expression matrices were subsequently processed using the limma package for normalisation and preprocessing, including probe/gene annotation, quality control and removal of outlier samples.

### 2.2 Identification of Differentially Expressed SUMOylation‐Related Oncogenes in HNSCC

Differentially expressed genes (DEGs) between HNSCC and nontumour controls in the TCGA cohort were identified using limma (version 3.54.0; https://bioconductor.org/packages/limma/). SUMOylation‐related genes were obtained from the GOBP_PROTEIN_SUMOYLATION gene set in Molecular Signatures Database (MSigDB). Oncogene‐related genes were retrieved from GeneCards using ‘oncogene’ as the search term. The intersection of (i) HNSCC DEGs (Supporting Information S2; Table S1), (ii) SUMOylation‐related genes (Supporting Information S2; Table S2), and (iii) oncogenes yielded the set of differentially expressed SUMOylation‐related oncogenes in HNSCC (Supporting Information S2; Table S3).

### 2.3 Machine Learning

We applied the least absolute shrinkage and selection operator (LASSO) algorithm using the glmnet package (version 4.1–7; https://github.com/cran/glmnet). A total of 522 HNSCC samples and 44 normal samples from TCGA were randomly divided into training and validation sets at a ratio of 7:3. The expression profiles of SUMOylation‐related oncogenes in the training set were used as input features to construct a discriminative model for distinguishing HNSCC tissues from normal tissues. The optimal penalty parameter was selected by cross‐validation, and genes with nonzero coefficients were retained to construct the final model. The model‐derived risk score was calculated as: Score = ∑CoefAi × expression of gene Ai. This score formula was then applied to all eligible datasets containing the complete set of model genes. Model robustness across cohorts was comprehensively evaluated using standardised mean difference (SMD) forest plots, receiver operating characteristic (ROC) curves, summary receiver operating characteristic (SROC) analysis, sensitivity‐specificity forest plots, positive likelihood ratio (PLR) and negative likelihood ratio (NLR) forest plots. The schematic workflow for construction and validation of the machine learning model in this study is shown in Supporting Information S1; Figure S1.

### 2.4 Functional Enrichment Analysis

Within the TCGA cohort, samples were stratified into high‐score and low‐score groups using the median score as the cutoff. DEGs between high‐score and low‐score groups were identified in the TCGA‐HNSCC dataset. Gene Ontology (GO) enrichment, Kyoto Encyclopaedia of Genes and Genomes (KEGG) pathway analysis and Gene Set Enrichment Analysis (GSEA) were performed using the clusterProfiler package (version 4.6.2; https://github.com/YuLab‐SMU/clusterProfiler) to functionally annotate these DEGs.

### 2.5 Somatic Mutation Analysis in HNSCC

Somatic mutation data for TCGA‐HNSCC were collected and analysed using the maftools (version 2.18.1; https://github.com/PoisonAlien/maftools) package to characterise mutation patterns of SUMOylation‐related oncogenes and to gain preliminary insights into the potential involvement of SUMOylation in HNSCC. In addition, we compared the mutational landscapes between the high‐score and low‐score groups.

### 2.6 Immune Infiltration and Immunotherapy Potential in HNSCC

The GSEABase (version 1.60.0; https://github.com/Bioconductor/GSEABase) package was used to quantify immune cell infiltration and immune‐related functional pathway activity in TCGA‐HNSCC samples. Differences in immune infiltration levels and immune functional pathway scores between the high‐score and low‐score groups were compared. In addition, immunophenotype data were obtained from The Cancer Immunome Atlas (TCIA), and differences in the immunophenoscore between high‐score and low‐score groups were evaluated to infer potential responsiveness to immunotherapy.

### 2.7 Collection, Preprocessing, and Analysis of scRNA‐Seq Data in HNSCC

#### 2.7.1 Data Acquisition, Quality Control and Dimensionality Reduction

Single‐cell RNA sequencing (scRNA‐seq) enables the characterisation of complex cellular states and provides a powerful approach for investigating disease‐associated cellular heterogeneity. Therefore, scRNA‐seq analysis was performed in this study [15]. ScRNA‐seq data from primary HNSCC and matched control tissues were obtained from the GEO database (GSE173468). Raw count matrices were processed using the Seurat package (version 4.4.0; https://github.com/satijalab/seurat). Briefly, low‐quality cells and genes were filtered according to standard quality‐control procedures, followed by data normalisation, scaling, identification of highly variable genes and principal component analysis (PCA). To reduce potential batch effects among samples, dataset integration and batch‐effect correction were performed using the Harmony package (version 1.2.0; https://github.com/immunogenomics/harmony). The top 20 principal components were used for clustering and uniform manifold approximation and projection (UMAP) visualisation.

#### 2.7.2 Cell Annotation and Identification of Malignant Epithelial Cells

Cell clusters were annotated using the SingleR package (version 2.0.0; https://github.com/dviraran/SingleR). To distinguish malignant epithelial cells from nonmalignant epithelial cells, copy number variation (CNV) inference was performed using the inferCNV package (version 1.14.2; https://github.com/broadinstitute/infercnv) with epithelial cells from matched control tissues used as the reference. Epithelial cells with inferred CNV alterations in HNSCC samples were regarded as malignant epithelial cells for subsequent analyses.

#### 2.7.3 SUMOylation‐Related Scoring, Proliferation Analysis and Cell‐Cycle Assessment

To investigate the potential association between SUMOylation‐related features and malignant epithelial cell behaviour, a cell‐level SUMOylation‐related signature score was calculated for each malignant epithelial cell based on the genes included in the SUMOylation‐related signature. Malignant epithelial cells were then divided into high‐score and low‐score groups according to the median signature score. Proliferation scores for malignant HNSCC cells were calculated using the AddModuleScore_UCell function in seurat (version 4.4.0; https://github.com/satijalab/seurat) package. Proliferation markers included *MKI67*, *IGF1*, *ITGB2*, *PDGFC*, *JAG1* and *PHGDH* [16]. Considering the ability of scRNA‐seq to resolve cell‐cycle states at single‐cell resolution, cell‐cycle phase assignment and cell‐cycle scoring were performed for malignant epithelial cells [17]. Comparisons between high‐score and low‐score malignant epithelial cells were performed using a mixed‐effects model with score group as a fixed effect and patient identity as a random intercept.

### 2.8 Integrated Analysis of Spatial Transcriptomics in HNSCC

#### 2.8.1 Data Acquisition and Preprocessing of Spatial Transcriptomics Data

Spatial transcriptomics data from primary HNSCC tissues were obtained from the GEO database (GSE281978). The gene expression matrix and corresponding spatial spot information were imported and processed using a workflow analogous to that used for scRNA‐seq analysis, including quality control, normalisation, dimensionality reduction, clustering and UMAP visualisation.

#### 2.8.2 Estimation of Cell‐Type Composition in Spatial Spots

Cell‐type composition of spatial spots was inferred using the Seurat package with the annotated scRNA‐seq dataset as the reference and the spatial transcriptomic dataset as the query. Cell‐type prediction scores for spatial spots were calculated using FindTransferAnchors and TransferData with SCT normalisation, the first 30 principal components and a k.weight value of 40 and were used to estimate the relative abundance of major cell populations in each spot. This approach allowed the spatial distribution of malignant epithelial cells and other cell populations within HNSCC tissues to be inferred.

#### 2.8.3 Spatial Mapping of the SUMOylation‐Related Signature

For each spatial spot, the expression levels of SUMOylation‐related genes were quantified, and spot level SUMOylation‐related signature scores were calculated based on the genes included in the established signature. The spatial distribution of these scores was then visualised across tissue sections. We further examined the relationship between spot‐level signature scores, malignant epithelial cell enrichment and the spatial localisation of SUMOylation‐related oncogene expression.

### 2.9 Evaluating the Proliferative Impact of Target Genes Across 72 HNSCC Cell Lines

To assess the contribution of target genes to HNSCC cell growth, CRISPR knockout screening data for 72 HNSCC cell lines were obtained from DepMap. Chronos scores were used as gene effect scores [18]. A gene effect score < −1 was considered indicative of a marked growth‐inhibitory phenotype upon gene knockout [19].

### 2.10 Cell Culture of HNSCC Cells and *SAE1* Knockdown

#### 2.10.1 Cell Culture

Human HNSCC cell lines SCC‐9 and SAS were purchased from the Cell Bank of the Chinese Academy of Sciences (Shanghai, China). SCC‐9 cells were cultured in DMEM/F12 medium (Gibco, USA), whereas SAS cells were maintained in DMEM (Gibco, USA). Both media were supplemented with 10% foetal bovine serum (FBS; Gibco, USA) and 1% penicillin‐streptomycin solution (P1400; Solarbio, China). Cells were maintained at 37°C in a humidified incubator with 5% CO_2_.

#### 2.10.2 siRNA‐Mediated Knockdown of Target Genes in SCC‐9 and SAS Cells

Small interfering RNA (siRNA) sequences were purchased from GenePharma (Suzhou, China). Two siRNAs targeting *SAE1* (si‐SAE1‐1 sense: 5′‐GAU UCG UAC UGG GUC UGU UTT‐3′, si‐SAE1‐1 antisense: 5′‐AAC AGA CCC AGU ACG AAU CTT‐3′; si‐SAE1‐2 sense: 5′‐GAU GCU GUG UGU CUG ACU UTT‐3′, si‐SAE1‐2 antisense: 5′‐AAG UCA GAC ACA CAG CAU CTT‐3′) and a negative control siRNA (si‐NC sense: 5′‐UUC UCC GAA CGU GUC ACG UTT‐3′, si‐NC antisense: 5′‐ACG UGA CAC GUU CGG AGA ATT‐3′) were transfected into SCC‐9 and SAS cells using Lipofectamine 3000 (L3000015; Thermo Fisher Scientific, USA) according to the manufacturer's instructions.

#### 2.10.3 Real‐Time Quantitative PCR

To knock down *SAE1* in SCC‐9 and SAS cells, siRNA transfection was performed as described above. Total RNA was extracted using the SevenFast Total RNA Extraction Kit (SM130; SevenBio, China). RNA concentration was measured with a NanoDrop One spectrophotometer (Thermo Fisher Scientific, USA). cDNA was synthesised using the TransScript Uni All‐in‐One First‐Strand cDNA Synthesis SuperMix (AU341; TransGen Biotech, China) following the manufacturer's protocols. RT‐qPCR was carried out on the QuantStudio 6 Real‐Time PCR System (Thermo Fisher Scientific, USA) using 0.2 mL 96‐well plates (Thermo Fisher Scientific, USA) and SYBR Green Mix (A25742; Thermo Fisher Scientific, USA). GAPDH served as the internal reference. Relative SAE1 mRNA expression was calculated using the 2^(‐ΔΔCt) method. Primers were synthesised by Sangon Biotech (China) as follows:

SAE1 forward: 5′‐TGG GTC TGT TGG CCG AAA TAG‐3′

SAE1 reverse: 5′‐ACA CAG CAT CGA ATT GAG TGA A‐3′

GAPDH forward: 5′‐TTG CCA TCA ATG ACC CCT TCA‐3′

GAPDH reverse: 5′‐CGC CCC ACT TGA TTT TGG A‐3′

#### 2.10.4 Western Blotting

Total proteins were extracted from SCC‐9 and SAS cells using RIPA lysis buffer. Equal amounts of protein were separated by SDS‐PAGE and transferred onto PVDF membranes. After blocking with 5% bovine serum albumin (BSA), the membranes were first incubated overnight at 4°C with anti‐SAE1 primary antibody (1:1000; R25653, ZenBio, China) followed by incubation with fluorescence‐conjugated secondary antibodies protected from light. The membranes were subsequently incubated with anti‐β‐tubulin primary antibody (1:5000; R380628, ZenBio, China) and the corresponding fluorescence‐conjugated secondary antibodies. Protein bands were visualised using a fluorescence imaging system and quantified with ImageJ software (version 1.54q). *β*‐tubulin was used as the internal loading control.

### 2.11 In Vitro Functional Assays

#### 2.11.1 Cell Viability Assay

Cell proliferation/viability was assessed using the Cell Counting Kit‐8 (CCK‐8). Briefly, 1000 transfected cells were seeded into 96‐well plates. At the indicated time points, 10 μL of CCK‐8 reagent (BS350A; Biosharp, China) was added to each well and incubated for 2 h at 37°C in a humidified incubator. Absorbance at 450 nm (OD450) was measured to quantify cell viability.

#### 2.11.2 Colony Formation Assay

A total of 1000 transfected HNSCC cells were plated in 12‐well plates and cultured for 10 days at 37°C with 5% CO_2_ and saturated humidity. Colonies were fixed with 4% paraformaldehyde (P1110; Solarbio, China) and stained with 0.1% crystal violet (G1062; Solarbio, China). Colony numbers in each well were quantified using ImageJ software (version 1.54q).

#### 2.11.3 Wound‐Healing Assay

Transfected HNSCC cells were seeded in 12‐well plates and cultured overnight until ∼90% confluence. A linear scratch was generated using a sterile pipette tip. Detached cells were removed by PBS washing, and images were captured immediately (0 h). Cells were then incubated at 37°C with 5% CO_2_, and images were acquired at 9 and 18 h to monitor migration. Wound area was measured using ImageJ software (version 1.54q). Wound closure rate was calculated as: Wound closure (%) = (Area_0h − Area_nh)/Area_0h × 100%.

#### 2.11.4 Cell Cycle Assay

The digested cell suspension was collected into centrifuge tubes and centrifuged at 1000 rpm for 5 min. After discarding the supernatant, the cells were washed with PBS. Cell cycle staining was then performed according to the manufacturer's instructions using a Cell Cycle Detection Kit (CCS012, MultiSciences (Lianke), China). Briefly, the cell pellets were resuspended with an appropriate volume of DNA staining solution and 10 μL of permeabilisation solution, mixed gently and incubated at room temperature in the dark for 30 min. Cell cycle distribution was detected using a CytoFLEX flow cytometer (Beckman Coulter, USA), and the data were analysed using ModFit LT software (version 5.0.9).

#### 2.11.5 Cell Apoptosis Assay

Cell apoptosis was assessed by flow cytometry using an Annexin V/PI Apoptosis Detection Kit (KTA0002, Abbkine, China). After cell culture, suspended cells and adherent cells digested with EDTA‐free 0.25% trypsin were collected and centrifuged at 2000 rpm for 5 min. The cell pellets were washed with pre‐cooled PBS and resuspended in 1 × Binding Buffer. According to the manufacturer's instructions, 2 μL Annexin V staining solution and 2.5 μL PI staining solution were added to each tube followed by incubation on ice in the dark for 15 min. Cell apoptosis was then detected using a CytoFLEX flow cytometer (Beckman Coulter, USA). Unstained, PI single‐stained and Annexin V single‐stained controls were used for parameter adjustment and compensation.

### 2.12 Statistical Analysis

All statistical analyses were performed using *R* (version 4.2.2), Stata (version 12.0) and GraphPad Prism (version 9.5.1). The *Q* test and *I* ^2^ statistics were used to determine the appropriate model for calculating SMD. When *I* ^2^ > 50%, substantial heterogeneity was assumed and a random‐effects model was applied; otherwise, a fixed‐effects model was used. For comparisons of means between two or multiple groups, t‐tests, one‐way ANOVA or rank‐sum tests were selected as appropriate based on data characteristics. All experiments were performed with three biological replicates. For both RT‐qPCR and CCK‐8 assays, each biological replicate was analysed with three technical replicates. A two‐sided *p* < 0.05 was considered statistically significant.

## 3 Results

### 3.1 Identification of Differentially Expressed SUMOylation‐Related Oncogenes in HNSCC

**FIGURE 2:**
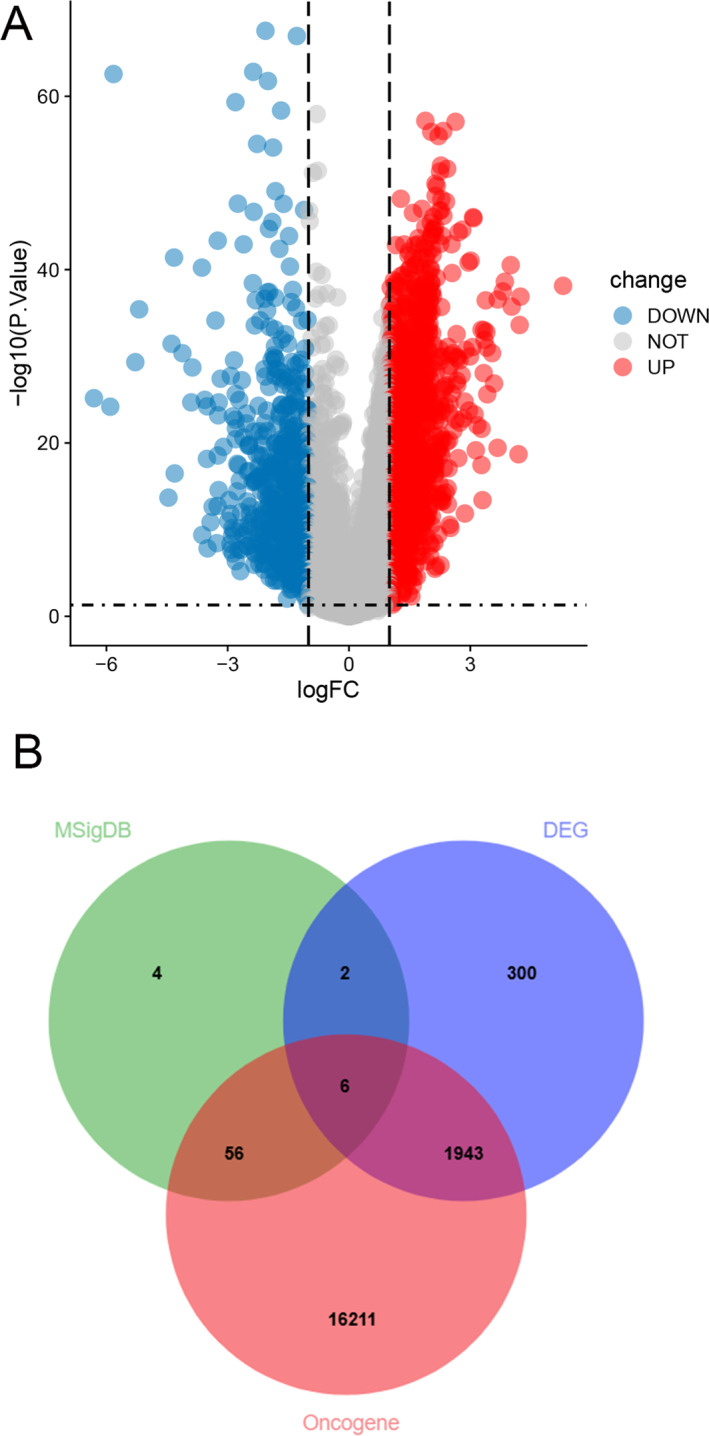
Identification of differentially expressed SUMOylation‐related oncogenes in the TCGA‐HNSCC cohort. (A) Differentially expressed genes (DEGs) identified in the TCGA‐HNSCC cohort. (B) Venn diagram showing the intersection of the MSigDB SUMOylation‐related gene set, the TCGA‐HNSCC DEG set and the GeneCards oncogene gene set.

Differential expression analysis between HNSCC and adjacent/normal control tissues in the TCGA cohort identified 1633 significantly upregulated genes and 618 downregulated genes (|logFC| > 1, *p* < 0.05) (Figure 2A). To pinpoint SUMOylation‐related oncogenic candidates, we intersected the DEG set with SUMOylation‐associated genes and curated oncogenes, yielding *EGR1*, *IFIH1*, *RANGAP1*, *RASD2* and *SAE1* as differentially expressed SUMOylation‐related oncogenes in HNSCC (Figure 2B).

### 3.2 LASSO‐Derived Recognition Signature and Multicohort Validation

**FIGURE 3:**
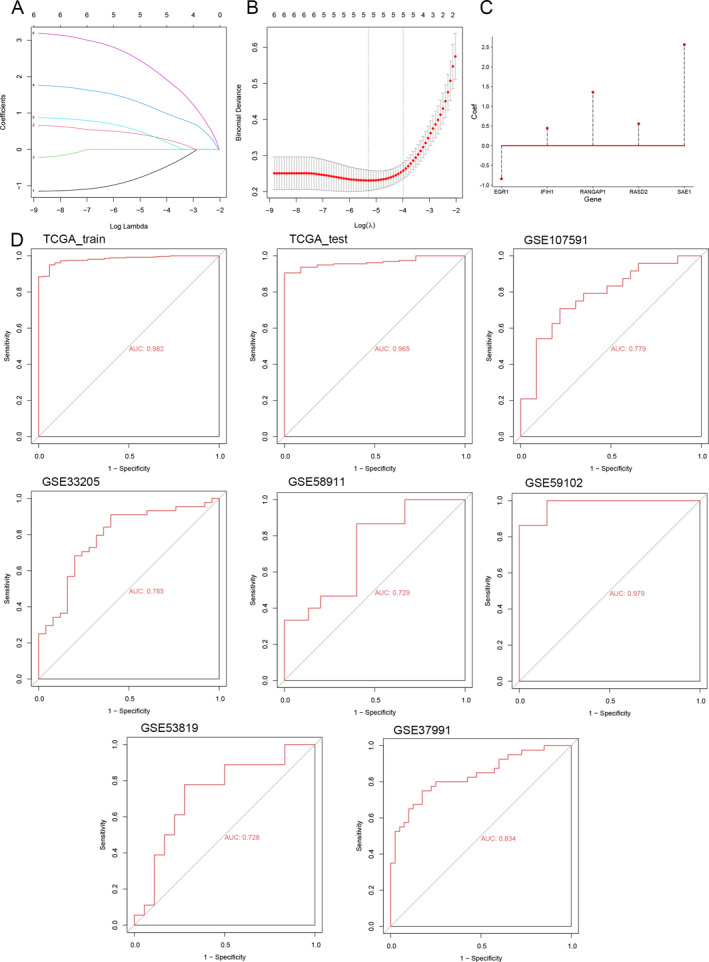
Construction and multicohort validation of a SUMOylation‐related oncogene–based recognition model for HNSCC. (A, B) LASSO regression used to develop the SUMOylation‐related oncogene recognition model for HNSCC. (C) Coefficients of the five hub genes retained in the SUMOylation‐related recognition model. (D) ROC curves evaluating model performance across eight cohorts, including the TCGA training set, TCGA internal validation set and GEO datasets GSE107591, GSE33205, GSE58911, GSE59102, GSE53819 and GSE37991.

To evaluate the clinical relevance of these differentially expressed SUMOylation‐related oncogenes, we applied LASSO regression for feature selection. In the TCGA training set, five genes were retained in the optimal model (Figure 3A,B). In the final signature, the coefficients were: *EGR1* (coef = −0.839), *IFIH1* (coef = 0.445), *RANGAP1* (coef = 1.358), *RASD2* (coef = 0.557) and *SAE1* (coef = 2.565) (Figure 3C). Based on this signature, scores were computed for all samples across the included cohorts. Using the median score in the TCGA cohort as the cutoff, samples were stratified into high‐score and low‐score groups. Comparison of clinicopathological characteristics between these groups showed that only N stage differed significantly (*p* < 0.05) (Supporting Information S1; Figure S2). To enhance clinical interpretability, a nomogram incorporating the five LASSO‐selected variables was constructed (Supporting Information S1; Figure S3). The recognition performance of the signature was subsequently validated in the TCGA training set, TCGA validation set and six external GEO cohorts. As shown in Figure 3D, the model achieved high discriminative accuracy across datasets: TCGA training (AUC = 0.982), TCGA validation (AUC = 0.965), GSE107591 (AUC = 0.779), GSE33205 (AUC = 0.785), GSE58911 (AUC = 0.729), GSE59102 (AUC = 0.979), GSE53819 (AUC = 0.728) and GSE37991 (AUC = 0.834).

**FIGURE 4:**
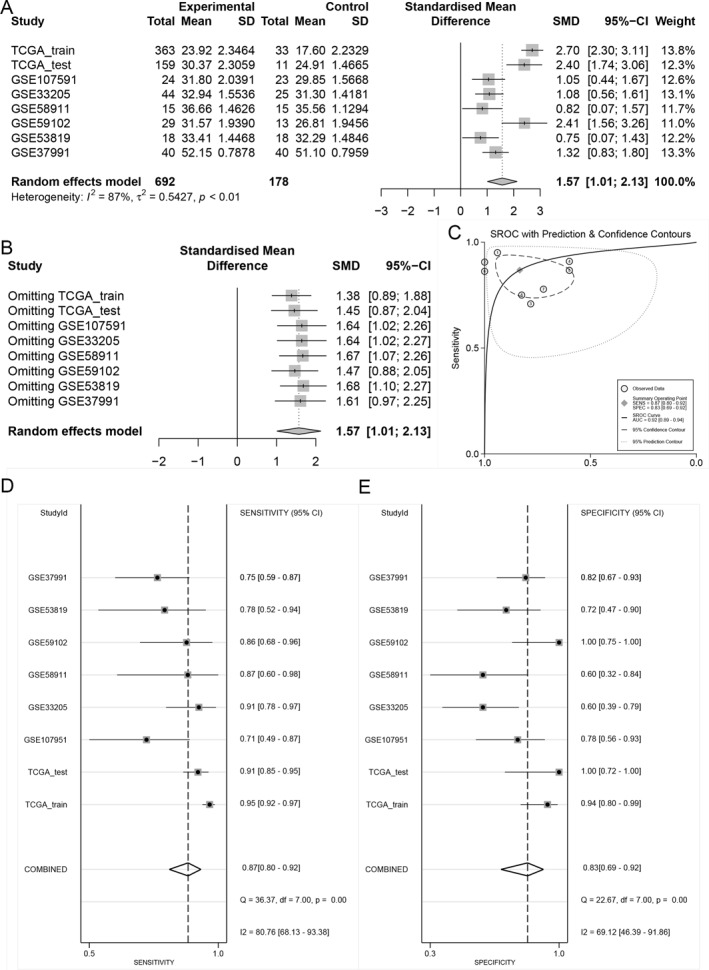
Integrated evaluation of the recognition accuracy of the SUMOylation‐related oncogene signature for HNSCC. (A) Combined comparison of model‐derived scores between HNSCC and non‐HNSCC samples across eight cohorts. (B) Leave‐one‐out sensitivity analysis shown as a forest plot. (C) Summary receiver operating characteristic (SROC) curve summarising the overall recognition performance of the signature across eight cohorts. (D) Forest plot of summary sensitivity estimates across eight cohorts. (E) Forest plot of summary specificity estimates across eight cohorts.

To comprehensively quantify the overall recognition utility, we performed an integrated multicohort analysis. Across eight cohorts, scores were significantly higher in 692 HNSCC samples than in 178 non‐HNSCC controls (SMD = 1.57, 95% CI 1.01–2.13, *p* < 0.05) (Figure 4A). Sensitivity analysis demonstrated that omission of any single cohort did not materially alter the pooled SMD (Figure 4B), supporting the robustness of the meta‐analytic estimate. From an aggregated perspective, the SROC curve (Figure 4C), sensitivity/specificity forest plots (Figure 4D,E), and likelihood‐ratio forest plot (Supporting Information S1; Figure S4) collectively indicated consistently strong recognition performance across cohorts (AUC = 0.92, SENS = 0.87, SPEC = 0.83, PLR = 5.24, NLR = 0.16). These findings demonstrate that the SUMOylation‐related recognition signature exhibits excellent and generalisable performance in both internal and external validations as well as in pooled multicenter analyses.

**FIGURE 5:**
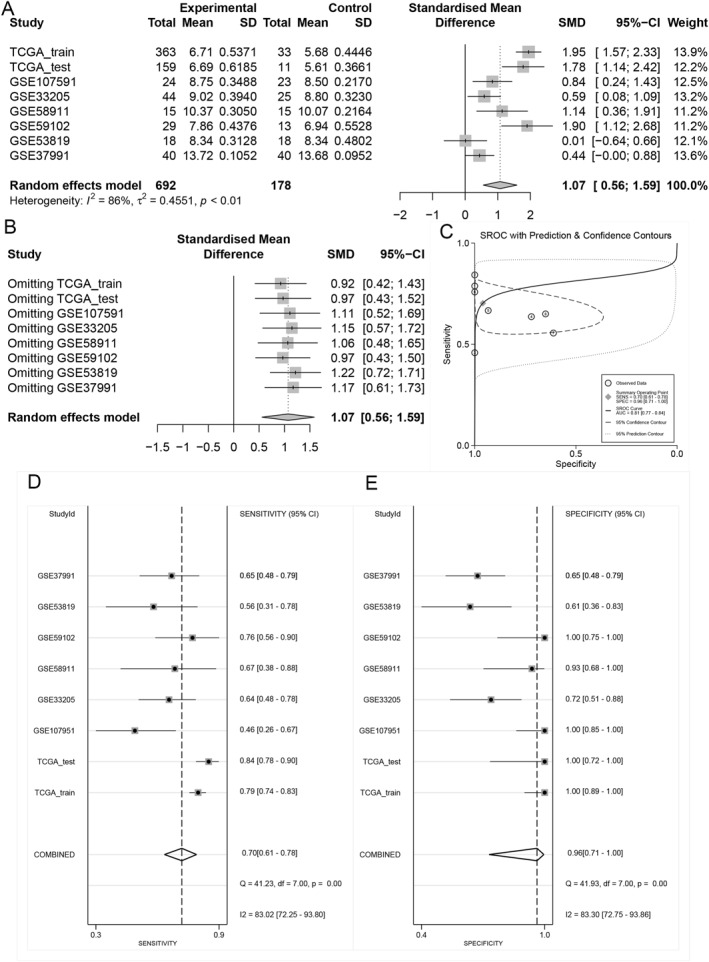
Integrated evaluation of *SAE1*, the key contributor to the SUMOylation‐related recognition model, for HNSCC identification. (A) Combined comparison of SAE1 expression levels between HNSCC and non‐HNSCC samples across eight cohorts. (B) Leave‐one‐out sensitivity analysis shown as a forest plot. (C) Summary receiver operating characteristic (SROC) curve summarising the overall accuracy of SAE1 for HNSCC identification across eight cohorts. (D) Forest plot of summary sensitivity estimates for *SAE1* across eight cohorts. (E) Forest plot of summary specificity estimates for *SAE1* across eight cohorts.

*SAE1* had strong contribution among the five features, we further assessed its standalone recognition value. *SAE1* expression was significantly higher in HNSCC tissues than in non‐HNSCC controls (SMD = 1.07, 95% CI 0.56–1.59, *p* < 0.05) (Figure 5A), and sensitivity analysis supported the stability of the pooled estimate (Figure 5B). Consistently, the SROC curve (Figure 5C), sensitivity/specificity forest plots (Figure 5D,E), and likelihood‐ratio forest plot (Supporting Information S1; Figure S5) indicated that *SAE1* alone retained strong discriminative capacity across the eight cohorts (AUC = 0.81, SENS = 0.70, SPEC = 0.96, PLR = 17.58, NLR = 0.31). Collectively, although *SAE1* alone did not outperform the full multigene signature, it nevertheless shows substantial potential as an HNSCC biomarker.

### 3.3 Pathway Level Differences Between SUMOylation‐Associated High‐Score Group and Low‐Score Group

**FIGURE 6:**
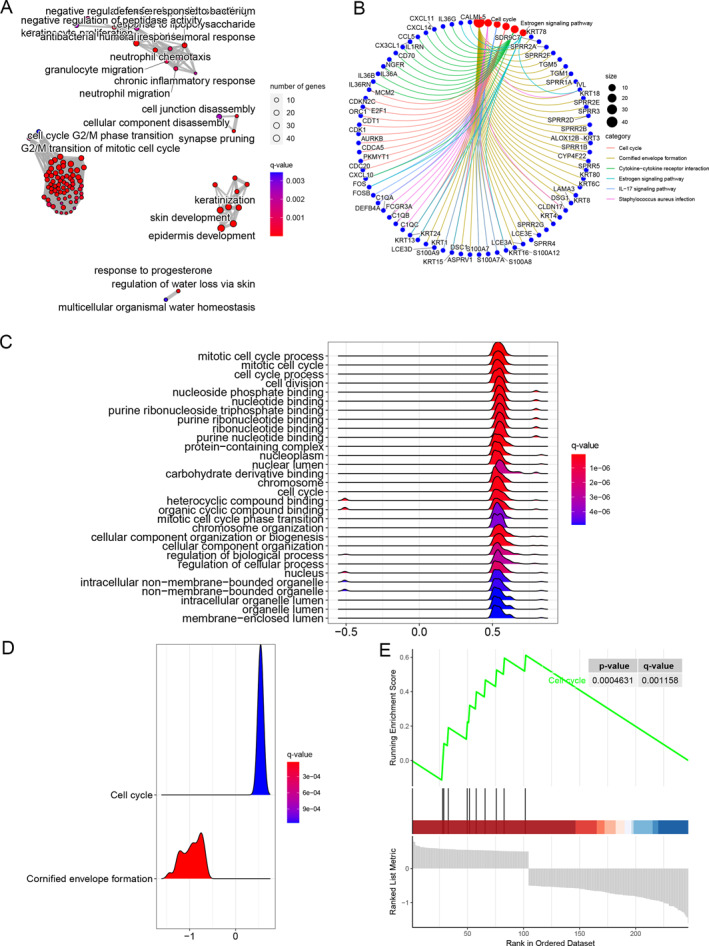
Functional enrichment analyses comparing the high‐score and low‐score groups. (A) Gene Ontology (GO) enrichment analysis of differentially expressed genes between high‐score and low‐score groups. (B) Kyoto Encyclopaedia of Genes and Genomes (KEGG) pathway enrichment analysis of differentially expressed genes between high‐score and low‐score groups. (C) Gene Set Enrichment Analysis (GSEA) of GO gene sets between high‐score and low‐score groups. (D) GSEA of KEGG gene sets between high‐score and low‐score groups. (E) GSEA highlighting enrichment of the cell cycle pathway within the KEGG gene sets.

The TCGA cohort was divided into the high‐score and low‐score groups using the median model score of 24.82 as the cutoff. Differential expression analysis identified 248 DEGs between the high‐score and low‐score groups in TCGA‐HNSCC (|logFC| > 0.5, *p* < 0.05). These DEGs were subsequently subjected to functional enrichment analyses. GO enrichment highlighted prominent associations with cell cycle G2/M phase transition, G2/M transition of mitotic cell cycle and chronic inflammatory response (Figure 6A). Consistently, KEGG analysis suggested that SUMOylation‐associated risk stratification was linked to pathways including cell cycle, IL‐17 signalling pathway and cytokine‐cytokine receptor interaction (Figure 6B). Notably, GSEA consistently highlighted cell‐cycle related gene sets (Figure 6C–E), corroborating the GO/KEGG results and implicating cell‐cycle dysregulation as a key axis distinguishing the two risk strata.

### 3.4 Mutational Landscape of HNSCC

As an exploratory analysis, we compared somatic mutation profiles between the high‐score and low‐score groups. Overall mutation frequencies were similar between the two groups (96.18% vs. 95.38%) (Supporting Information S1; Figure S6A,B). Missense mutations were the predominant variant class, and C > T transitions were the most frequent base substitution pattern. Mutation frequencies of the five hub SUMOylation‐related genes were low in both groups (Supporting Information S1; Figure S6C,D).

### 3.5 Immune Microenvironment Features Associated With SUMOylation‐Related Risk Groups

**FIGURE 7:**
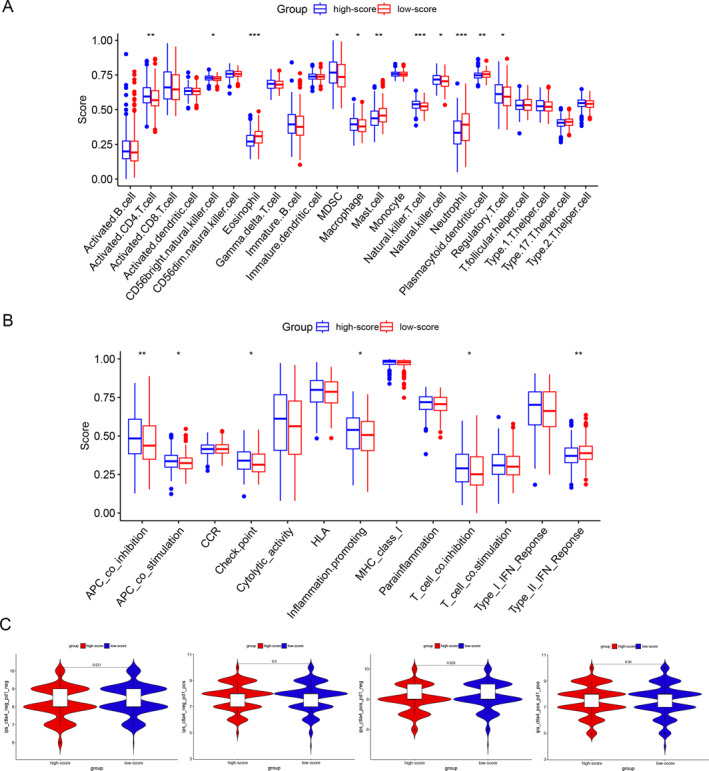
Immune microenvironment features associated with the SUMOylation‐related score in HNSCC. (A) Comparison of immune cell infiltration levels between the high‐score and low‐score groups. (B) Comparison of immune‐related functional pathway scores between the high‐score and low‐score groups. (C) Comparison of PD1 and CTLA4 stratified immunophenoscores between the high‐score and low‐score groups. *p < 0.05; **p < 0.01; ***p < 0.001; ****p < 0.0001.

To characterise the immune contexture of HNSCC, we performed immune infiltration analysis in TCGA‐HNSCC. As shown in Figure 7A, infiltration scores of multiple immune cell subsets (e.g., Activated B cell, Activated CD4^+^ T cell) differed between high‐score and low‐score groups with several cell types exhibiting statistically significant differences (*p* < 0.05). In parallel, immune functional signatures (e.g., APC_co_inhibition, APC_co_stimulation) also displayed distinct activity patterns across risk groups, and a subset of immune functions was significantly different between strata (*p* < 0.05) (Figure 7B). These findings indicate that SUMOylation‐associated risk stratification captures heterogeneity in both immune cell infiltration and immune functional state. Notably, all immune‐cell infiltration features (Supporting Information S2; Table S4) and immune‐function scores (Supporting Information S2; Table S5) with nominal between‐group differences met an exploratory FDR‐adjusted threshold of *q* < 0.10, indicating that these immune‐related differences were relatively robust.

Consistently, immunophenotype analysis showed significant differences in immunophenoscore (IPS) between high‐ and low‐score groups (*p* < 0.05) notably in IPS PD1 (−) CTLA4 (−) and IPS PD1 (−) CTLA4 (+) categories (Figure 7C). Together, these results suggest that SUMOylation‐related markers may have potential utility for predicting immunotherapy responsiveness in HNSCC.

### 3.6 Single‐Cell Landscape of HNSCC

**FIGURE 8:**
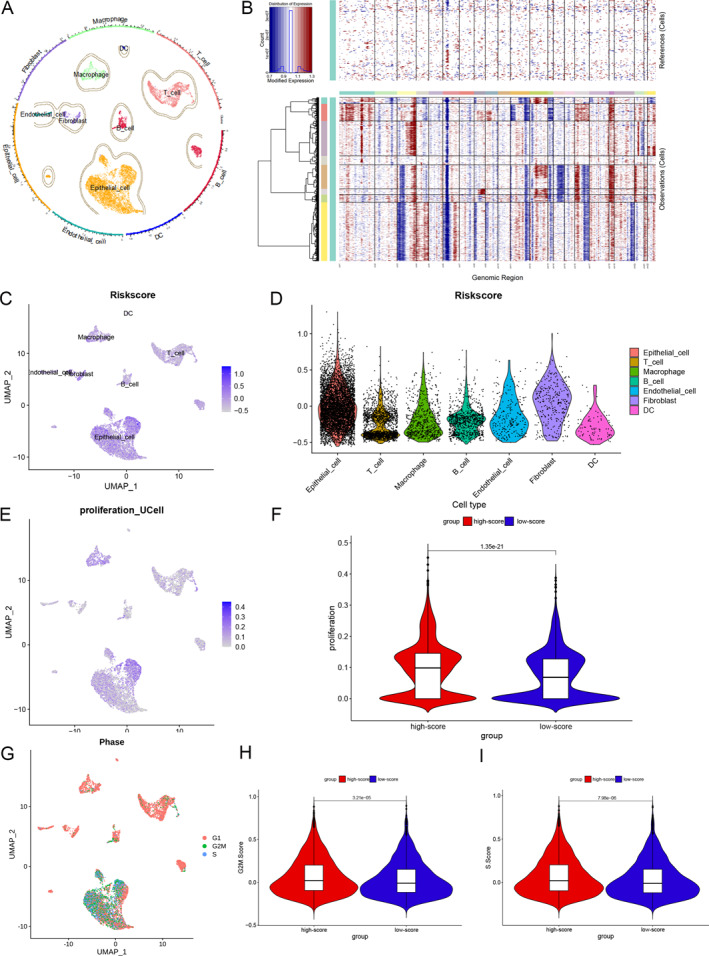
Single‐cell transcriptomics further validates the role of the SUMOylation‐related score in HNSCC. (A) Identification of major cell types in HNSCC tissues. (B) Malignancy assessment of epithelial cells in HNSCC using control tissues as the reference. (C) Distribution of the SUMOylation‐related score in HNSCC. (D) Comparison of score levels across major cell types in HNSCC. (E) Proliferation scores across major cell types in HNSCC. (F) Comparison of proliferation scores between high‐score and low‐score malignant epithelial cells. *p* values were calculated using the mixed‐effects model. (G) Cell‐cycle phase assignment across cells in HNSCC. (H) Comparison of G2M scores between high‐score and low‐score malignant epithelial cells. *p* values were calculated using the mixed‐effects model. (I) Comparison of S scores between high‐score and low‐score malignant epithelial cells. *p* values were calculated using the mixed‐effects model.

Single‐cell analysis delineated seven major cell compartments in HNSCC tissues, including epithelial cells, endothelial cells, B cells, T cells, fibroblasts, dendritic cells (DCs) and monocyte‐macrophages (Figure 8A). Using control tissues as a reference, epithelial cells identified in tumour samples were further characterised as malignant epithelial cells (Figure 8B).

We next evaluated whether the SUMOylation‐related genes showed cell type specific enrichment. A signature score was computed across cell types using AddModuleScore_UCell based on these five genes and was defined as the SUMOylation‐related signature score (Figure 8C). Malignant epithelial cells and fibroblasts exhibited relatively higher scores, whereas other cell types showed lower scores (Figure 8D). These findings suggested that the signature was preferentially associated with tumour‐related cellular compartments particularly malignant epithelial cells.

We therefore focused on malignant epithelial cells to examine whether a higher SUMOylation‐related signature score was linked to enhanced proliferative activity. Malignant epithelial cells were subsequently stratified into high‐score and low‐score subsets using the median score. Proliferation scoring across cell types (Figure 8E) revealed that malignant epithelial cells in the high‐score subset had significantly higher proliferation scores than those in the low‐score subset (*p* < 0.05 and *q =* 4.06 × 10^−21^ < 0.05) (Figure 8F). Given the consistent enrichment of cell cycle related programs at the bulk level, we further assessed cell cycle activity in malignant epithelial cells at single cell resolution (Figure 8G). Both the G2M score (*q =* 3.21 × 10^−5^ < 0.05) (Figure 8H) and S score (*q =* 1.20 × 10^−5^ < 0.05) (Figure 8I) differed significantly between high‐score and low‐score malignant epithelial cells (*p* < 0.05). Collectively, these single‐cell results reinforce a potential role of SUMOylation‐related genes in shaping cell‐cycle‐associated programs within HNSCC. Collectively, these scRNA‐seq results indicate that the SUMOylation‐related signature is associated with proliferative and cell cycle activity in malignant epithelial cells.

### 3.7 Spatial Transcriptomic Profiling of HNSCC Tissues

**FIGURE 9:**
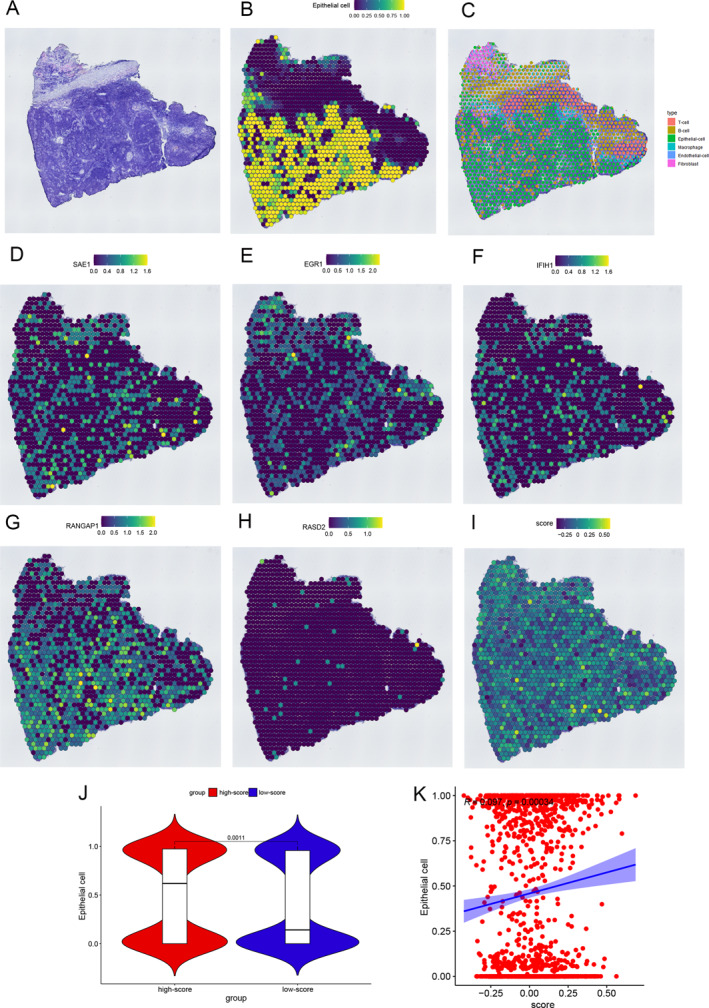
Spatial distribution of the SUMOylation‐related score in HNSCC. (A) Haematoxylin and eosin (HE)–stained section of HNSCC tissue. (B) Relative abundance of malignant epithelial cells across spots in the HNSCC section. (C) Estimated proportions of major cell types across spots in the HNSCC section. (D–H) Expression patterns of *SAE1* (D), *EGR1* (E), *IFIH1* (F), *RANGAP1* (G) and *RASD2* (H) across spots in the HNSCC section. (I) Score across spots in the HNSCC section. (J) Comparison of malignant epithelial cell abundance between high‐score and low‐score spots. (K) Correlation between spot‐level score and malignant epithelial cell abundance in the HNSCC section.

To further examine whether the SUMOylation‐related features showed spatial organisation within HNSCC tissues, spatial transcriptomic analysis was performed on an HNSCC tissue section (Figure 9A). Leveraging the cell‐type annotations derived from scRNA‐seq, we conducted deconvolution of the stRNA‐seq data to estimate the relative abundance of malignant epithelial cells in each spot (Figure 9B) along with the proportions of other major cell populations (Figure 9C).

To delineate the spatial landscape of SUMOylation in HNSCC, we further mapped the spot‐wise expression of the five hub SUMOylation‐related genes, including *IFIH1*, *EGR1*, *RASD2*, *SAE1* and *RANGAP1*, across the tissue section (Figure 9D–H). These genes exhibited heterogeneous spatial expression patterns, suggesting spatially structured SUMOylation‐associated programs within the tumour microenvironment. Based on these five hub genes, we computed a spot‐level score using the AddModuleScore_UCell algorithm (Figure 9I). Spots were then dichotomised into high‐score and low‐score groups using the median score across all spots in the section. Notably, high‐score spots displayed a significantly higher relative proportion of malignant epithelial cells than low‐score spots (*p <* 0.05 and *q* < 0.05) (Figure 9J). Moreover, the spot‐level score showed a modest but statistically significant correlation with the malignant epithelial cell fraction (*R* = 0.097, *p* < 0.05) (Figure 9K). Together, these results suggest a spatial association between the SUMOylation‐related signature score and the estimated abundance of malignant epithelial cells in this HNSCC tissue section.

### 3.8 Functional Validation of *SAE1* in HNSCC Cell Phenotypes

**FIGURE 10:**
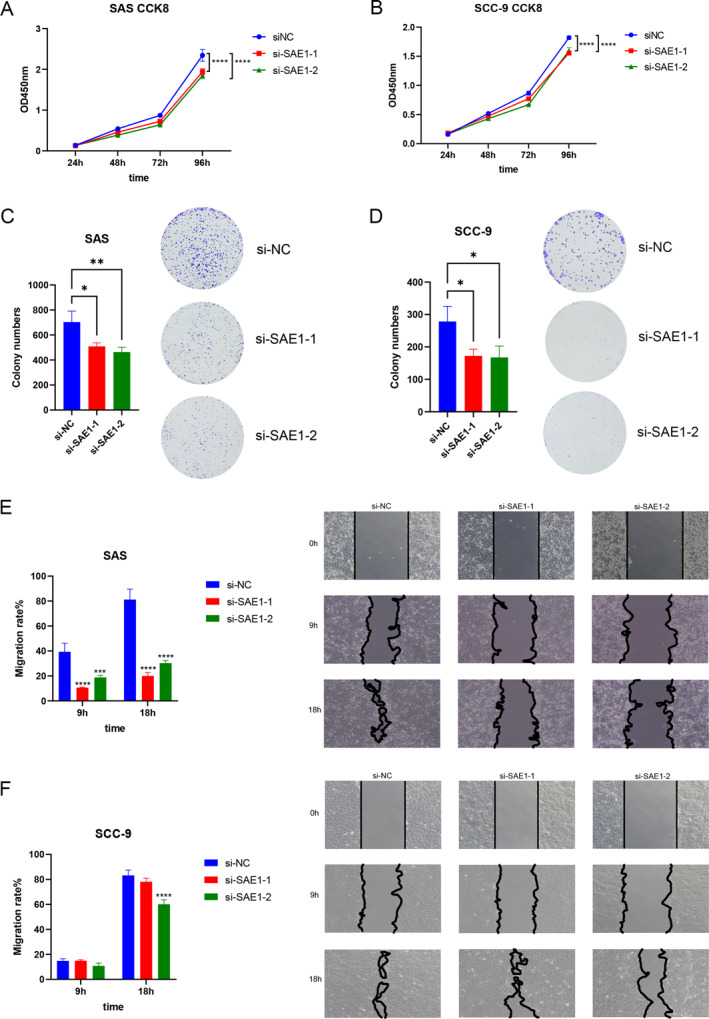
*SAE1* promotes proliferation and migration of HNSCC cells. CCK‐8 assays revealed that SAE1 silencing significantly reduced cell viability in SAS (A) and SCC‐9 (B) cells; Clonogenic potential of *SAE1*‐silenced SAS (C) and SCC‐9 (D) cells was evaluated by colony formation assays. Wound‐healing assays showed that *SAE1* knockdown impaired the migratory ability of SAS (E) and SCC‐9 (F) cells. Statistical significance: *p < 0.05, **p < 0.01, ***p < 0.001, ****p < 0.0001.

**FIGURE 11:**
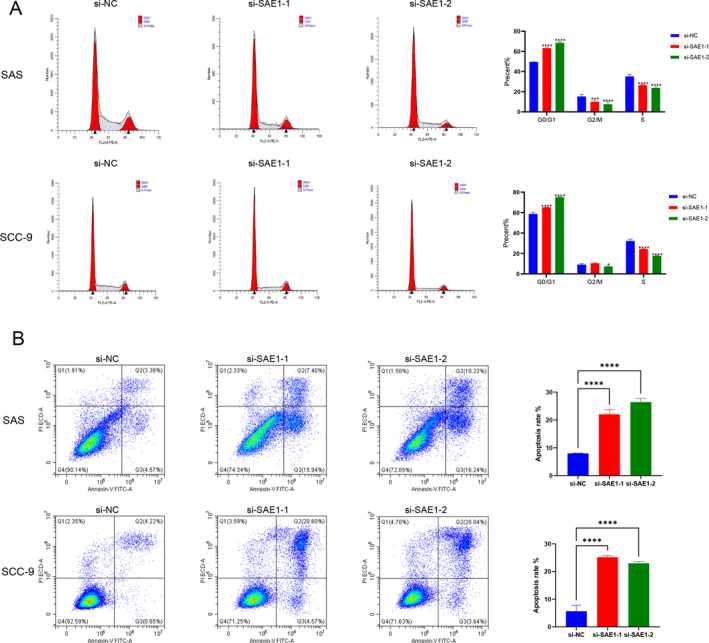
Regulation of cell cycle and apoptosis by *SAE1* in HNSCC cells (A) cell cycle analysis; (B) apoptosis analysis. Statistical significance: *p < 0.05, **p < 0.01, ***p < 0.001, ****p < 0.0001.

Given that *SAE1* had strong contribution among the five features and emerged as a promising candidate biomarker, we prioritised *SAE1* for experimental validation of its role in regulating HNSCC cellular behaviours, including proliferation and migration, in SAS and SCC‐9 cells. The knockdown efficiency of si‐SAE1 was confirmed by RT‐qPCR and Western blotting, which showed significantly reduced *SAE1* expression at the mRNA (Figure S7A–B) and protein levels (Figure S7C–D) in SAS and SCC‐9 cells (*p* < 0.05).CCK‐8 assays demonstrated a marked decrease in proliferative viability upon *SAE1* silencing in both cell lines (Figure 10A,B) (*p* < 0.05). Consistently, colony formation assays showed that *SAE1* knockdown significantly impaired clonogenic ability in SAS (Figure 10C) and SCC‐9 cells (Figure 10D) (*p* < 0.05). In addition, DepMap CRISPR screening data revealed that 67 of 72 (93%) HNSCC cell lines exhibited Chronos scores < −1 (Supporting Information S1; Figure S8), further supporting the requirement of *SAE1* for HNSCC cell growth. In wound‐healing assays, SCC‐9 cells transfected with si‐SAE1‐1 showed a trend towards reduced closure capacity (Figure 10F), although the difference did not reach statistical significance (*p* > 0.05). Across the remaining transfected groups, *SAE1* silencing consistently resulted in significantly reduced wound closure, indicating diminished migratory capacity (Figure 10E‐F) (*p* < 0.05). Cell cycle analysis further demonstrated that *SAE1* knockdown markedly decreased the S‐phase population and increased the G0/G1‐phase population in both cell lines (Figure 11A) (*p* < 0.05). Consistently, apoptosis analysis showed that *SAE1* knockdown significantly increased the apoptotic rate of SAS and SCC‐9 cells (Figure 11B) (*p* < 0.05). Therefore, *SAE1*, a key gene within the SUMOylation‐related signature established in this study, may play an important role in promoting HNSCC cell proliferation and migration as well as regulating cell‐cycle progression.

## 4 Discussion

Recently, some studies have further emphasised that HNSCC is a biologically heterogeneous disease comprising distinct molecular and HPV‐related contexts [20, 21, 22]. For example, recent integrative multi‐omics analyses have identified molecular subtypes of HNSCC with distinct biological features and therapeutic vulnerabilities, whereas proteogenomic profiling of HPV‐negative HNSCC has revealed complex alterations involving copy‐number drivers, phosphoproteomic signalling, RNA‐processing programs and tumour microenvironment‐related features [20, 21]. In parallel, HPV‐positive HNSCC has also been shown to contain biologically distinct subgroups, indicating that HPV status alone may not fully capture the molecular diversity of this disease [22]. In addition to these established molecular frameworks, several studies have suggested that SUMOylation‐related regulation may also be involved in head and neck squamous malignancies. In oral squamous cell carcinoma (OSCC), high‐throughput sequencing analyses have reported dysregulated expression of multiple SUMOylation regulators, including UBA2, UBE2I and SENP family members, and proposed SUMOylation‐related regulators as potential prognostic biomarkers [23]. Pharmacological inhibition of SUMOylation by ginkgolic acid was also shown to suppress OSCC progression by alleviating SUMOylation of SMAD4 [24]. In HNSCC, SENP1 has recently been reported to regulate ferroptosis and tumour progression through modulation of ACSL4 SUMOylation [25]. These studies support the biological relevance of SUMOylation‐related regulation in HNSCC but most focused on individual regulators or specific pathways, whereas an integrated SUMOylation‐related signature and its functional validation remain insufficiently explored.

In this study, we characterised SUMOylation‐related gene expression in HNSCC and established a SUMOylation‐associated recognition signature with robust cross‐cohort performance. Integrated analyses suggested that this signature was associated with cell‐cycle dysregulation, while in vitro experiments supported the functional role of *SAE1* in promoting HNSCC cell proliferation and migration. These findings indicate that SUMOylation‐associated pathways may contribute to HNSCC progression and warrant further mechanistic and translational investigation.

Given their quantitative, reproducible nature and suitability for external validation across independent cohorts, multigene signatures have become a prevailing paradigm for molecular diagnosis and risk stratification in HNSCC [26]. Here, we assembled a multicenter transcriptomic compendium comprising 870 head and neck tissue samples and leveraged machine learning for feature selection and model construction. The resulting SUMOylation‐related signature exhibited consistent discriminative capacity in independent validation cohorts, underscoring its robustness and potential generalisability. At the gene level, accumulating evidence suggests that several signature constituents exert coherent biological roles across HNSCC subtypes. *EGR1* is frequently downregulated in oral squamous cell carcinoma and has been shown to suppress proliferation, migration, and invasion [27]. In tongue squamous cell carcinoma, *EGR1* is similarly reduced and reportedly inhibits migratory and invasive phenotypes via transcriptional regulation of *DUSP1* [28]. In oropharyngeal carcinoma, *IFIH1* has been reported to be significantly upregulated at both mRNA and protein levels; mechanistically, *IFIH1* binds *PTTG1* mRNA to enhance its translation, thereby promoting proliferation and clonogenicity in Cal27/FaDu cells, whereas *IFIH1* knockdown suppresses proliferation and synergises with the *PTTG1* inhibitor PHA‐848125 to inhibit xenograft growth and prolong survival [29]. *RANGAP1* is also markedly upregulated in tumour tissues and associated with unfavourable prognosis; functional assays indicate that *RANGAP1* promotes proliferation, migration/invasion and metastatic dissemination potentially through METTL3/YTHDF1‐mediated m6A regulation and downstream CRABP2–MAPK signalling [30]. *RASD2* has been linked to malignant phenotypes in certain cancers; for example, *RASD2* silencing suppresses growth, migration and invasion in uveal melanoma [31]. Notably, *SAE1*, the E1 activating enzyme in the SUMOylation cascade, has been shown in other tumour contexts to drive aggressive behaviour by enhancing mTOR SUMOylation or facilitating p27 nuclear export, yet direct functional evidence in HNSCC remains relatively limited [32, 33]. Collectively, prior work supports broad oncogenic relevance of the signature genes, which strengthens the biological plausibility and interpretability of our model as a molecular discriminative tool for HNSCC. Nevertheless, much of the current evidence is derived from pan‐cancer studies or specific HNSCC subsites, and additional analyses in HNSCC cohorts will be required to further establish context‐specific mechanisms and validate clinical utility.

To further delineate the molecular functions of SUMOylation in HNSCC, we performed functional enrichment analyses, which consistently converged on cell cycle‐related pathways. The single‐cell landscape provided additional resolution, showing that within the malignant epithelial compartment, the high‐score subset exhibited significantly higher proliferation scores than the low‐score subset, accompanied by elevated G2M and S‐phase scores. These findings indicate that the risk states captured by our signature are tightly coupled to accelerated cell‐cycle progression. Our observations align well with prior evidence. Pharmacological inhibition of SUMO E1 with TAK‐981 markedly reduces global SUMOylation and induces cell cycle arrest, mitotic failure and chromosome segregation defects, thereby suppressing tumour cell proliferation. Similarly, E1 inhibition has been shown to trigger cell cycle blockade and chromatin bridge formation underscoring the requirement of SUMOylation for mitotic integrity and genomic stability [34]. From a genetic perspective, loss of *UBC9* causes a global abrogation of SUMOylation, leading to mitotic arrest and chromosome mis‐segregation and ultimately disrupting cell‐cycle progression [35]. Mechanistically, the SUMO–NIP45 axis converts toxic DNA catenanes into signals that activate the G2 checkpoint, thereby preventing cytokinesis failure and binucleation [36]. In addition, Tof2 polySUMOylation is cell‐cycle regulated and facilitates mitotic exit by promoting Cdc14 release [37]. Notably, both inhibition and aberrant stabilisation of mitotic SUMOylation can compromise chromosome segregation, highlighting the need for dynamic and reversible control of SUMOylation during mitosis [38]. Collectively, these studies support the notion that dysregulated SUMOylation can reshape cell‐cycle control and enhance proliferative capacity.

Emerging evidence further links SUMOylation to immune regulation. A recent study reported that an upstream transcription factor‐driven increase in *SAE1* promotes p53 SUMOylation and alters its functional state, thereby facilitating cell‐cycle entry and attenuating CD8^+^ T cell‐mediated immune clearance [39]. Concordantly, we observed significant differences between risk strata in multiple immune‐cell infiltration scores and immune functional pathway activities, and several immunophenoscore subcategories differed significantly between the high‐ and low‐score groups. These findings suggest that SUMOylation may contribute to immune modulation with potential implications for immunotherapy benefit.

In addition, stRNA‐seq analysis revealed that high‐score spots harboured a significantly higher relative abundance of malignant epithelial cells, and spot‐level scores showed a modest but significant correlation with malignant epithelial cell proportion. This indicates that SUMOylation‐associated high‐score states may exhibit spatial enrichment within tumour sections and co‐localise with malignant cell–dense regions. Given that SUMOylation can enhance stress adaptation and regulate transcriptional and proteostatic homoeostasis, these high‐score spatial niches may preferentially support rapid proliferation, invasion/migration and therapeutic resistance. Similar observations have been reported in other malignancies, where upregulated *SAE1* promotes hepatocellular carcinoma cell proliferation [40], and drives nonsmall cell lung cancer metastasis by facilitating epithelial–mesenchymal transition through N‐cadherin SUMOylation [41].

As the core initiator of SUMOylation and the most prominent contributor to our signature, *SAE1* emerged as the central candidate gene in this study. Given that its functional role in HNSCC remains largely unexplored, we further performed in vitro validation and demonstrated that *SAE1* substantially promotes proliferative viability, migratory capacity and clonogenic potential, while also regulating cell‐cycle progression in HNSCC cell lines. These findings are consistent with prior pan‐cancer evidence. In hepatocellular carcinoma, *SAE1* knockdown suppresses cell proliferation and migration, increases the proportion of cells in the G0/G1 phase and decreases that in the S phase, which is associated with the regulation of mTOR signalling via SUMOylation [32]. In haematologic malignancy models and related systems, *SAE1* has also been implicated in malignant phenotypes by influencing key cell‐cycle regulators, for instance, facilitating p27 nuclear export to evade growth suppression [33]. In the context of HNSCC, emerging clues likewise support a functional role for the SUMO axis: the UBC9/SUMO pathway participates in the processing of epithelial adhesion molecules and shapes cell motility phenotypes, whereas SENP1‐mediated SUMO‐dependent regulation can facilitate tumour progression [42]. Our results further strengthen the notion that SUMOylation exerts functionally relevant, potentially driving effects in HNSCC. Moreover, CRISPR screening data indicate that *SAE1* is broadly required for the growth of most HNSCC cell lines. Collectively, we propose that *SAE1* is likely a key hub within the SUMOylation network in HNSCC and may serve as a biomarker to prioritise for mechanistic interrogation and for exploring potential targeted interventions [43].

Despite these advances, several limitations warrant consideration and should be addressed in future work. First, although our HNSCC discriminative model demonstrated strong recognition performance and stability across multicenter cohorts validation in prospective clinical samples remains necessary. Second, the scRNA‐seq and stRNA‐seq datasets were relatively limited in sample size and may not fully capture the breadth of HNSCC heterogeneity. Third, although stRNA‐seq suggested the presence of spatial risk niches, these observations require replication across additional specimens and anatomical regions. Future studies integrating multi‐sample, multi‐region spatial validation, coupled with spatial proteomics and in situ assays, will be essential to more precisely localise *SAE1*‐SUMOylation‐active regions and to resolve their cell–cell interaction architectures. Fourth, this study mainly focused on shared SUMOylation‐related biological features across HNSCC. However, biological differences among HNSCC tumours arising from different anatomical subsites remain an important issue for further investigation. Future studies with larger cohorts and additional experimental validation are needed to further confirm and refine our findings.

## 5 Conclusions

We developed a SUMOylation‐related recognition model for HNSCC and demonstrated its robust performance across multiple external validation cohorts. The potential biological relevance of SUMOylation in HNSCC appears to be closely linked to dysregulated cell‐cycle pathways. *SAE1* may contribute to HNSCC progression by promoting cell proliferation and migration and regulating cell‐cycle progression, thereby representing a potential molecular biomarker for HNSCC.

## Author Contributions

**Zhe Fang:** conceptualization, methodology, software, formal analysis, data curation, visualization, writing – original draft. **Kai Mei:** methodology, software, formal analysis, data curation, visualization, writing – original draft. **Jiaqi Liu:** investigation, validation, formal analysis. **Wei Zhou:** investigation, validation, formal analysis. **Tingjing Li:** investigation, validation, formal analysis. **Hai Zhang:** conceptualization, supervision, project administration, writing – review and editing. **Chuangjie Cao:** conceptualization, supervision, project administration, writing – review and editing. All authors have read and approved the final manuscript.

## Funding

The authors have nothing to report.

## Ethics Statement

The authors have nothing to report.

## Consent

The authors have nothing to report.

## Conflicts of Interest

The authors declare no conflicts of interest.

## Supporting information


Supporting Information S1

Supporting Information S2

## Data Availability

The data that support the findings of this study are available from the corresponding author upon reasonable request.
