# Supplementary material for: Machine Learning Modelling, Single‐Cell Landscape Profiling and Spatial Transcriptomics Provide New Insights Into SUMOylation in Head and Neck Squamous Cell Carcinoma
Source: IET Syst Biol. 2026 Jul 29;20(1):e70082. doi: 10.1049/syb2.70082 (PMC13420368; doi:10.1049/syb2.70082)
Supplement: Supplementary file 1 — Supporting Information S1 [file SYB2-20-e70082-s002.docx]

**Supplementary Materials**

**
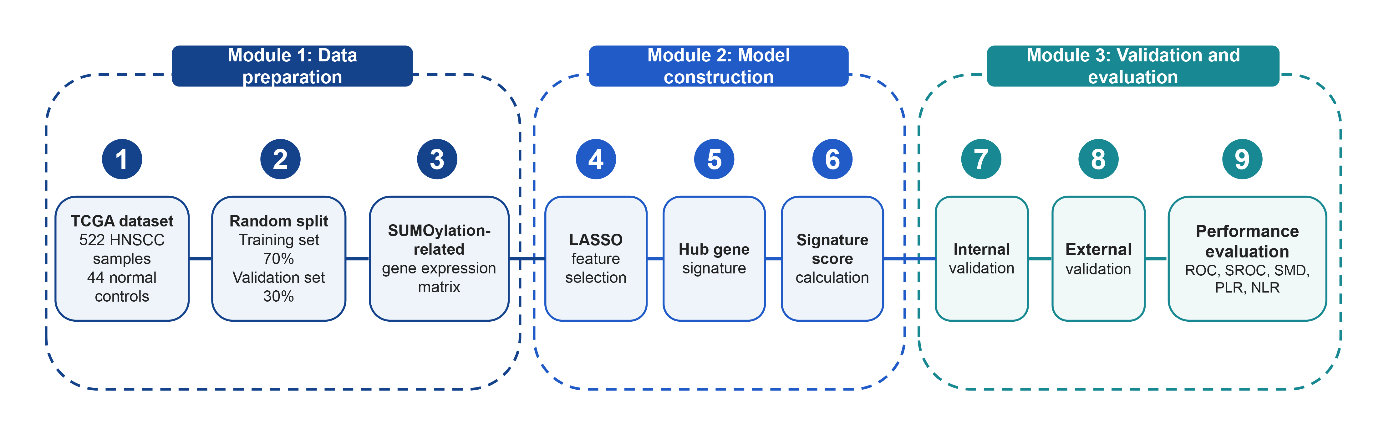
**

**Figure S1.** **Workflow for the construction and validation of the LASSO-based machine learning model.**


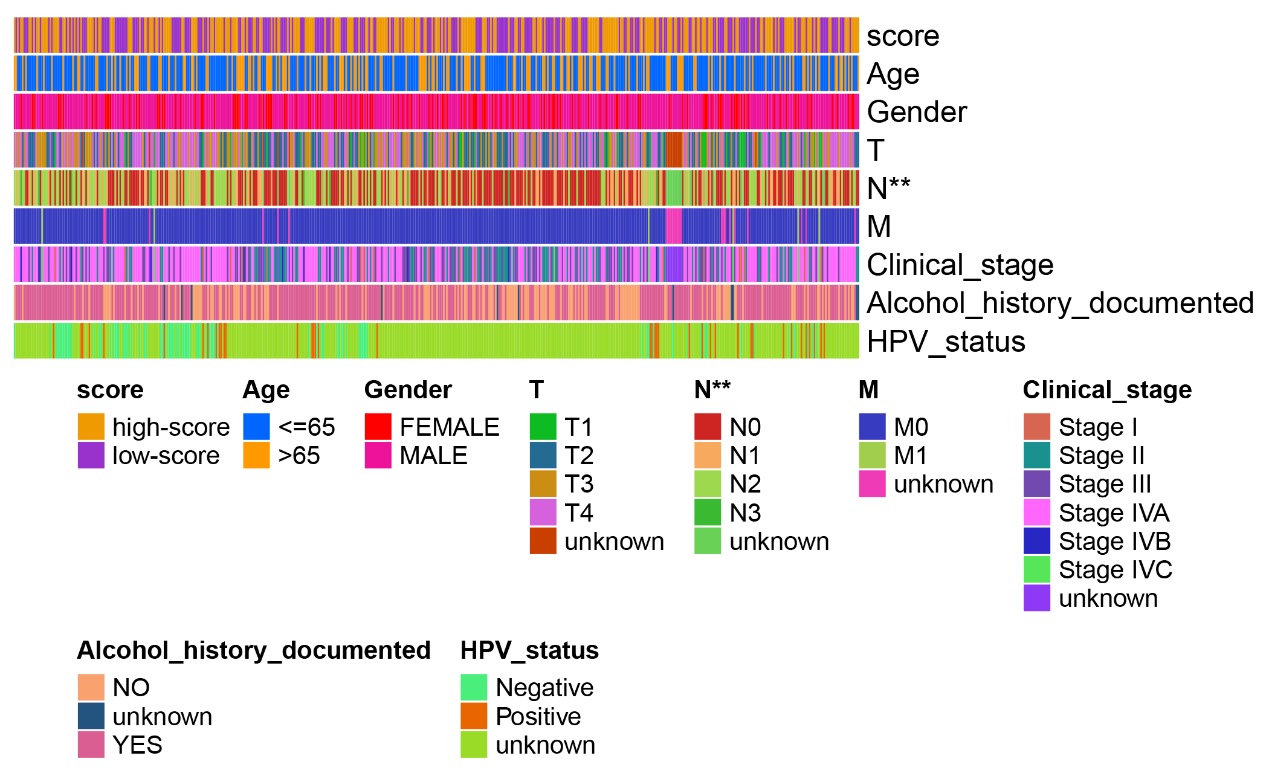


**Figure S2. Heatmap of clinicopathological characteristics in the TCGA cohort stratified by high-score and low-score groups.**

*: *p*<0.05; **: *p*<0.01; ***: *p*<0.001; ****: *p*<0.0001


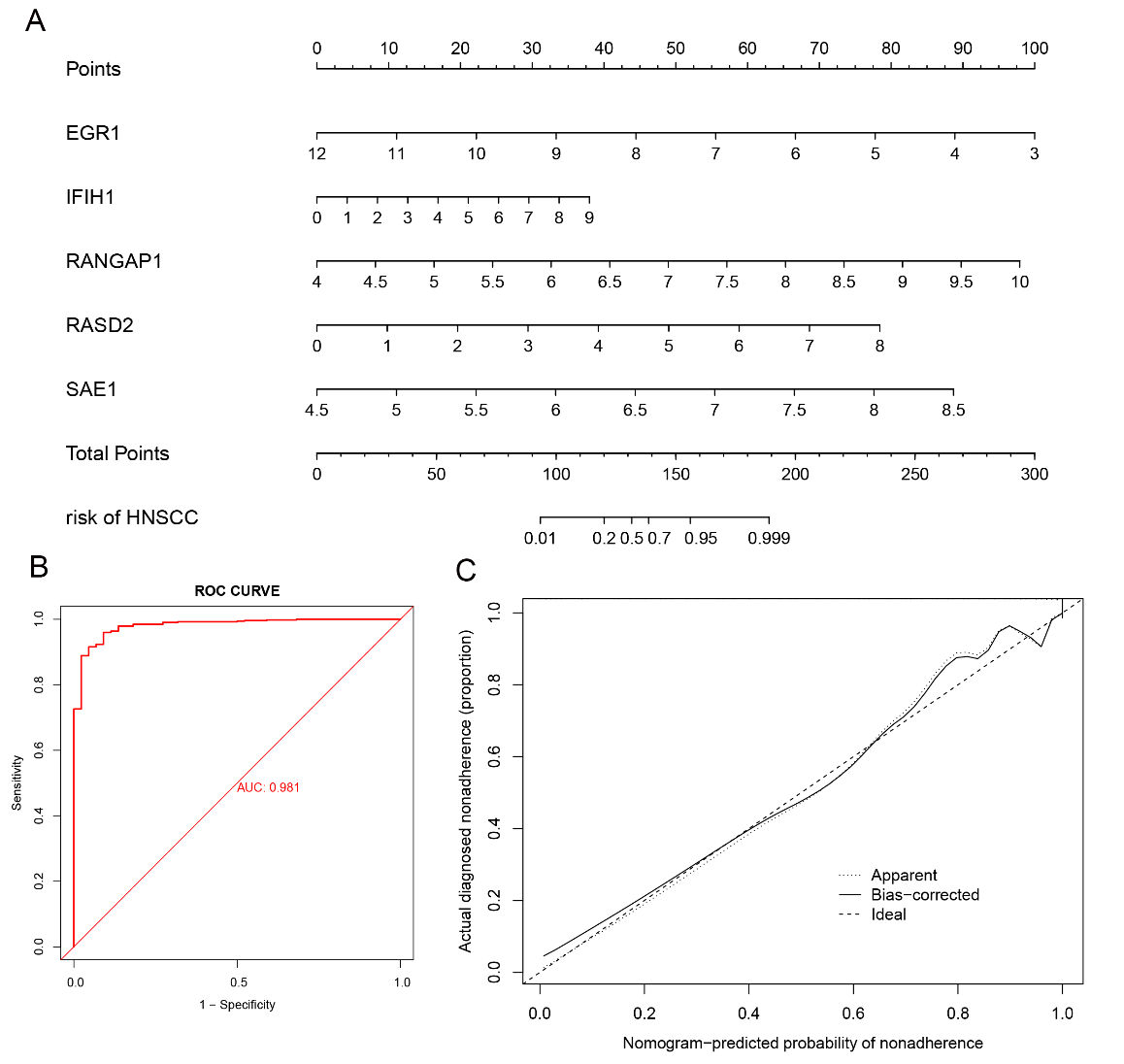


**Figure S3. Nomogram for the TCGA-HNSCC cohort.**
(A) Nomogram incorporating five hub SUMOylation-related genes to estimate the probability of HNSCC.
(B) ROC curve evaluating the identification performance of the nomogram for HNSCC probability.
(C) Calibration curve assessing the agreement between nomogram-estimated and observed probabilities of HNSCC.
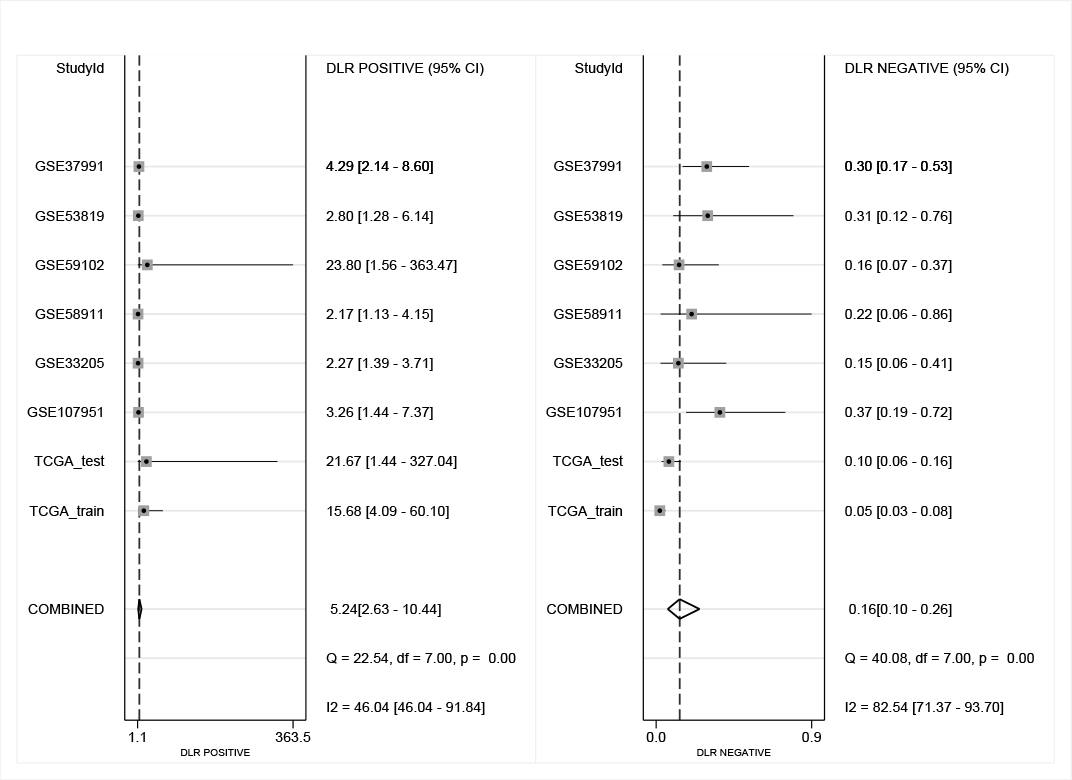


**Figure S4. Forest plots of positive and negative likelihood ratios for the SUMOylation-related score across eight HNSCC cohorts.**


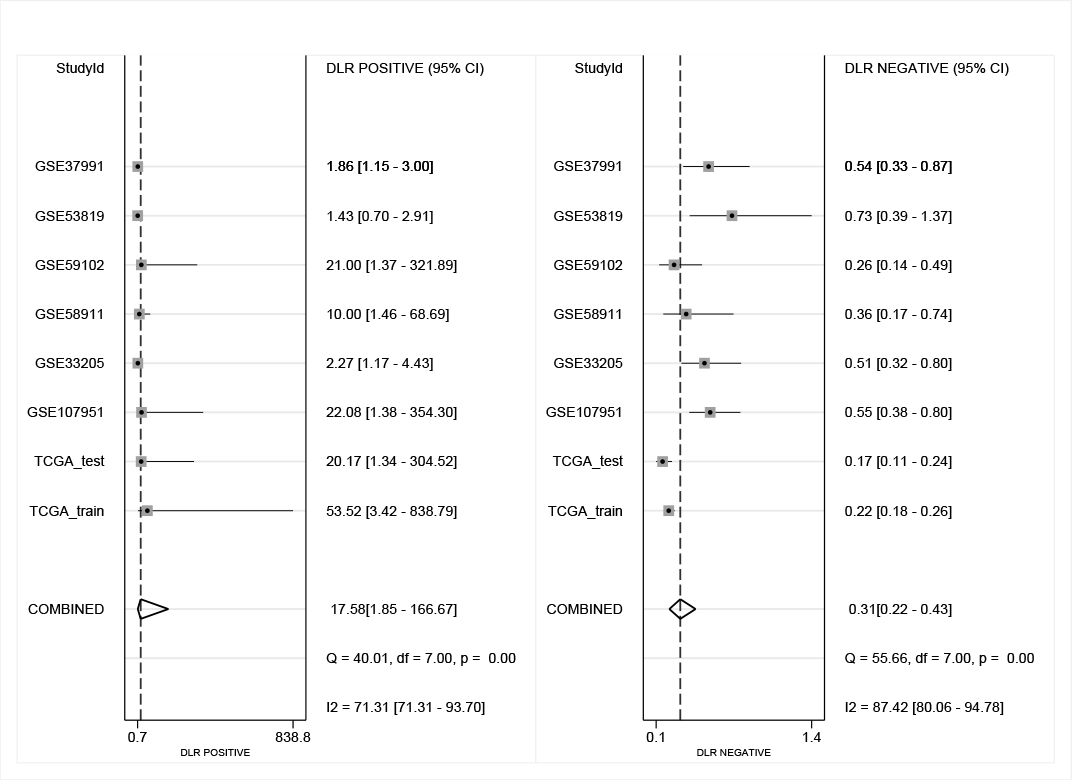


**Figure S5. Forest plots of positive and negative likelihood ratios for *SAE1* across eight HNSCC cohorts.**


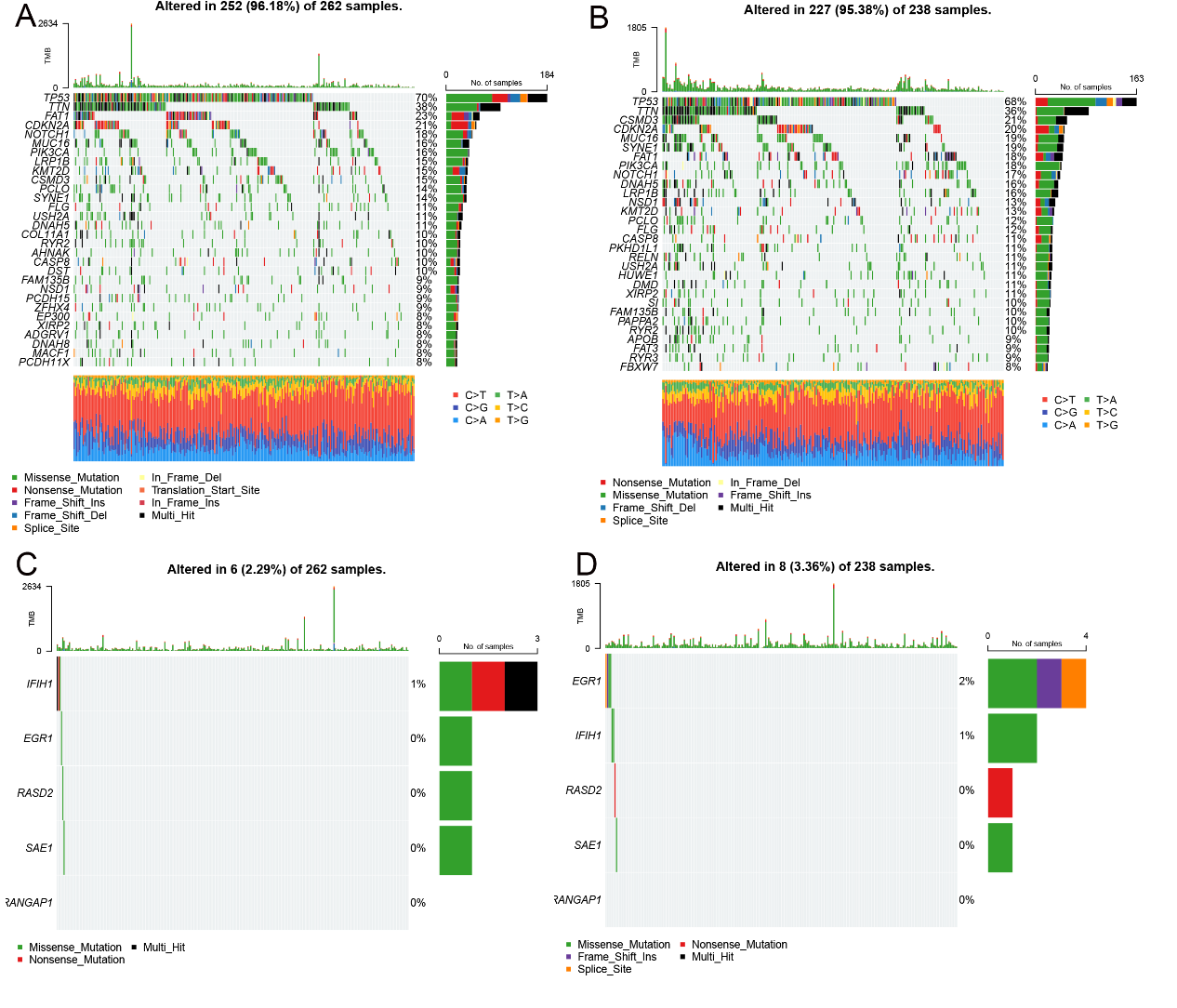


**Figure S6. Somatic mutational landscape of HNSCC stratified by SUMOylation-associated risk groups.**
(A) Top 30 most frequently mutated genes in the high-score group.
(B) Top 30 most frequently mutated genes in the low-score group.
(C) Mutation status of the five hub SUMOylation-related genes in the high-score group.
(D) Mutation status of the five hub SUMOylation-related genes in the low-score group.

**
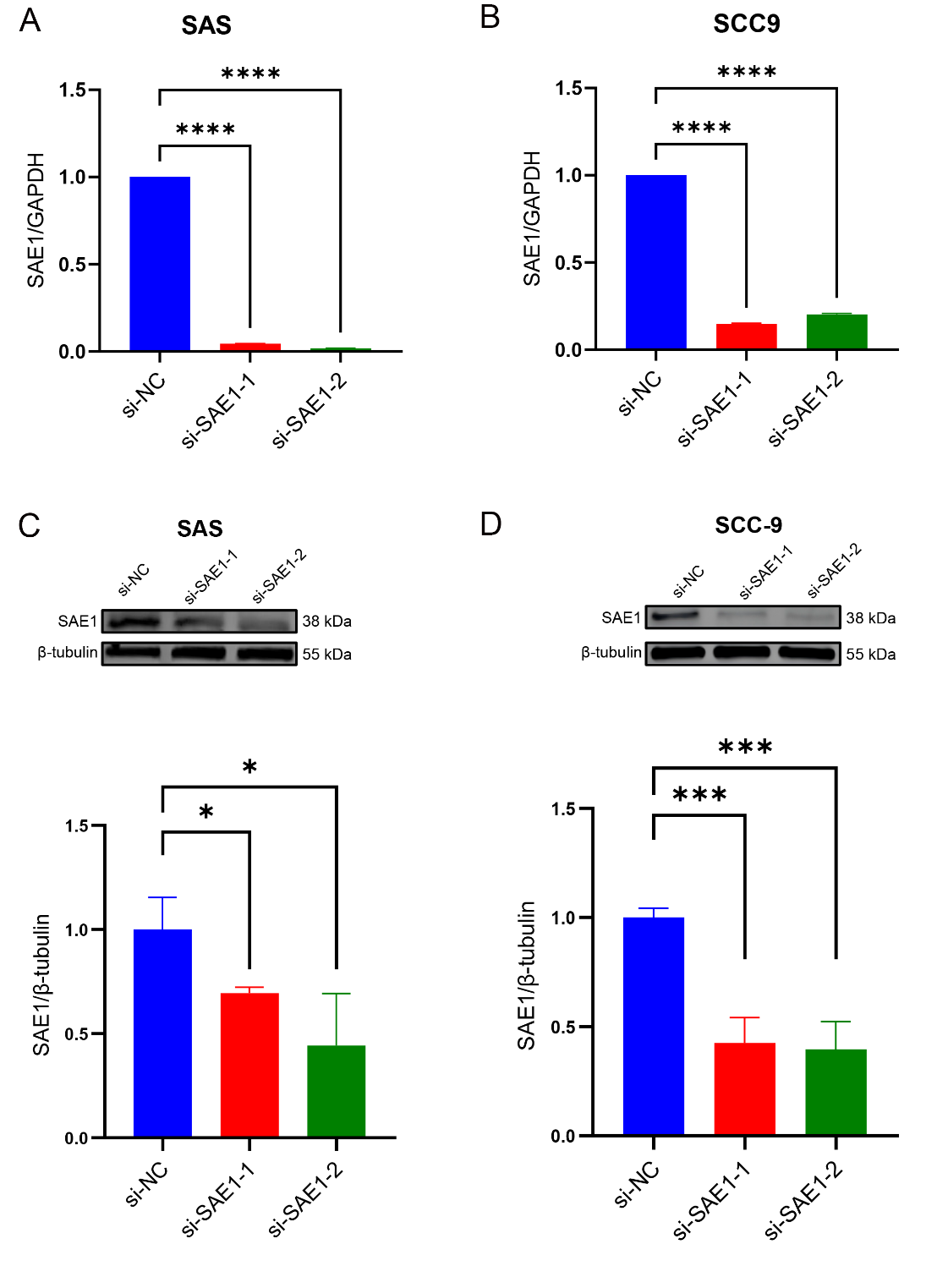
**

**Figure S7.** **Validation of *SAE1* knockdown efficiency in HNSCC cells at the mRNA and protein levels**

RT-qPCR confirmed the knockdown efficiency of *SAE1* after siRNA transfection in SAS (A) and SCC-9 (B) cells；

Western blot validated the knockdown efficiency of *SAE1* after siRNA transfection in SAS (C) and SCC-9 (D) cells.

Statistical significance:**p* < 0.05，***p* < 0.01，****p* < 0.001，*****p* < 0.0001.


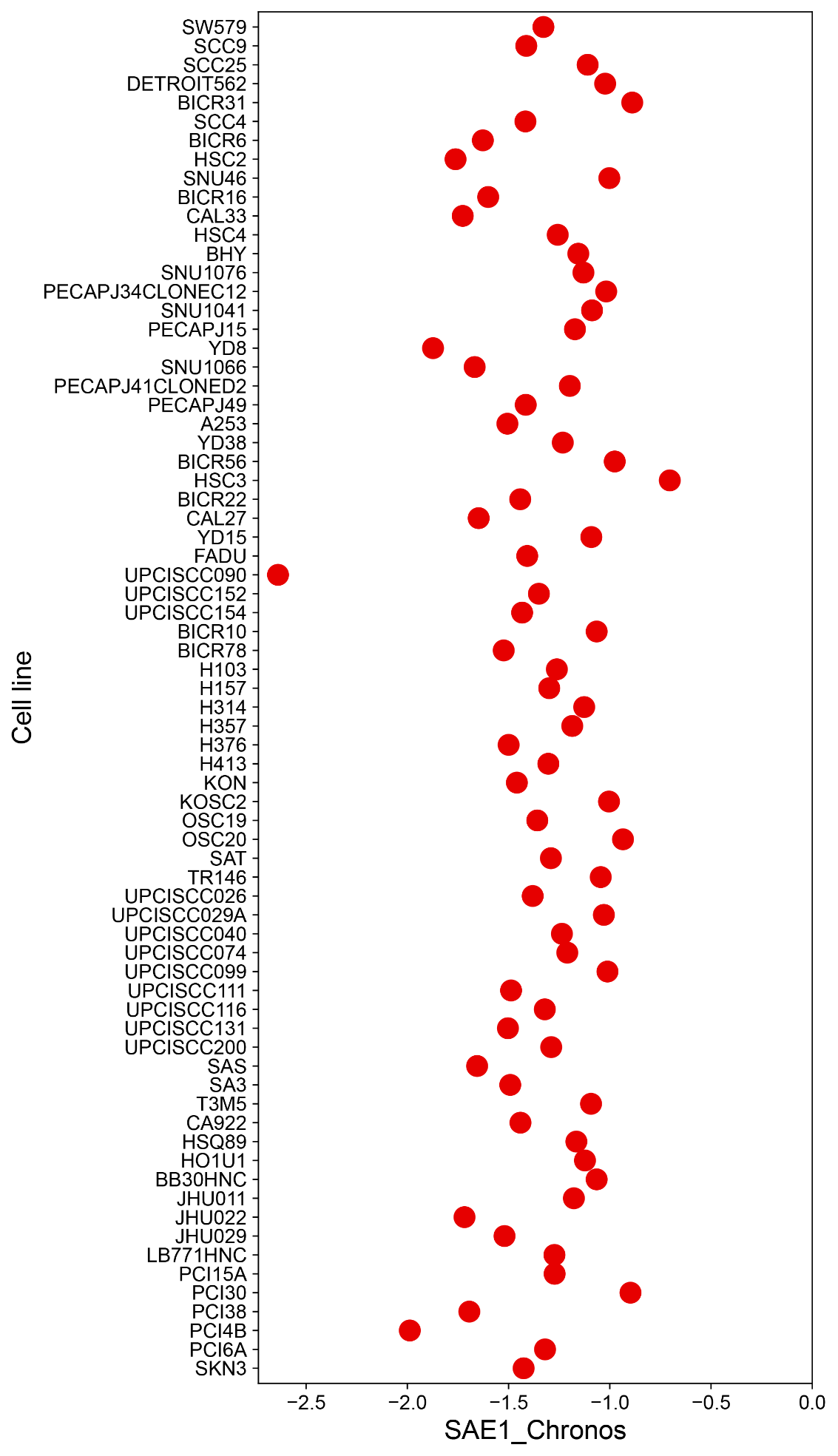


**Figure S8. Chronos scores of *SAE1* across HNSCC cell lines.**
